# Integrating machine learning with electronic health record data to facilitate detection of prolactin level and pharmacovigilance signals in olanzapine-treated patients

**DOI:** 10.3389/fendo.2022.1011492

**Published:** 2022-10-13

**Authors:** Xiuqing Zhu, Jinqing Hu, Tao Xiao, Shanqing Huang, Dewei Shang, Yuguan Wen

**Affiliations:** ^1^ Department of Pharmacy, The Affiliated Brain Hospital of Guangzhou Medical University, Guangzhou, China; ^2^ Guangdong Engineering Technology Research Center for Translational Medicine of Mental Disorders, Guangzhou, China; ^3^ Department of Clinical Research, Guangdong Second Provincial General Hospital, Guangzhou, China

**Keywords:** machine learning, prolactin, olanzapine, electronic health record, hyperprolactinemia, pharmacovigilance, XGBoost, SHAP

## Abstract

**Background and aim:**

Available evidence suggests elevated serum prolactin (PRL) levels in olanzapine (OLZ)-treated patients with schizophrenia. However, machine learning (ML)-based comprehensive evaluations of the influence of pathophysiological and pharmacological factors on PRL levels in OLZ-treated patients are rare. We aimed to forecast the PRL level in OLZ-treated patients and mine pharmacovigilance information on PRL-related adverse events by integrating ML and electronic health record (EHR) data.

**Methods:**

Data were extracted from an EHR system to construct an ML dataset in 672×384 matrix format after preprocessing, which was subsequently randomly divided into a derivation cohort for model development and a validation cohort for model validation (8:2). The eXtreme gradient boosting (XGBoost) algorithm was used to build the ML models, the importance of the features and predictive behaviors of which were illustrated by SHapley Additive exPlanations (SHAP)-based analyses. The sequential forward feature selection approach was used to generate the optimal feature subset. The co-administered drugs that might have influenced PRL levels during OLZ treatment as identified by SHAP analyses were then compared with evidence from disproportionality analyses by using OpenVigil FDA.

**Results:**

The 15 features that made the greatest contributions, as ranked by the mean (|SHAP value|), were identified as the optimal feature subset. The features were gender_male, co-administration of risperidone, age, co-administration of aripiprazole, concentration of aripiprazole, concentration of OLZ, progesterone, co-administration of sulpiride, creatine kinase, serum sodium, serum phosphorus, testosterone, platelet distribution width, α-L-fucosidase, and lipoprotein (a). The XGBoost model after feature selection delivered good performance on the validation cohort with a mean absolute error of 0.046, mean squared error of 0.0036, root-mean-squared error of 0.060, and mean relative error of 11%. Risperidone and aripiprazole exhibited the strongest associations with hyperprolactinemia and decreased blood PRL according to the disproportionality analyses, and both were identified as co-administered drugs that influenced PRL levels during OLZ treatment by SHAP analyses.

**Conclusions:**

Multiple pathophysiological and pharmacological confounders influence PRL levels associated with effective treatment and PRL-related side-effects in OLZ-treated patients. Our study highlights the feasibility of integration of ML and EHR data to facilitate the detection of PRL levels and pharmacovigilance signals in OLZ-treated patients.

## Introduction

Prolactin (PRL), a polypeptide hormone, is primarily synthesized in and secreted from the anterior pituitary gland, and plays multiple roles in lactation, reproduction, and organ homeostasis (1). PRL secretion is regulated by stimulatory factors like the thyrotropin-releasing hormone (TRH) and inhibitory factors like dopamine (DA) in the hypothalamus, and is influenced by alterations in both physiological (e.g., pregnancy, stress, and sleep states) and pathological conditions (e.g., pituitary disorders, central nervous system disorders, and systemic diseases) (2, 3). Hyperprolactinemia is commonly defined as the condition of a sustained increase in PRL up to that of a fasting level (at least 2 h after waking) of above 20 ng/mL (~424 mIU/L) in men, and above 25 ng/mL (~530 mIU/L) in women (4). It has been found to be associated with an increased risk of many diseases, such as cardiovascular mortality in males (5), cancer (6), bone loss, and fractures (7). In particular, psychiatric patients with hyperprolactinemia usually exhibit short-term sexual dysfunction, amenorrhoea or galactorrhoea, and long-term sequelae such as osteoporosis (8). Recently, a nationwide study in Finland demonstrated that the long-term use of PRL-increasing antipsychotics is significantly associated with the increased risk of breast cancer in females with schizophrenia (9). Studies *in vivo* and *in vitro* have revealed that hyperprolactinemia-inducing antipsychotics can prompt precancerous lesions to progress to cancer *via* activating JAK-STAT5 (10).

A large group of drugs, including psychotropic drugs like antipsychotics, have the potential to cause the hypersecretion of PRL, which is the most common pharmacological cause of hyperprolactinemia (3, 11). In both short- and long-term toxicological studies with rodents, the drug-induced mechanisms of hypo- and hyperprolactinemia commonly involve the dopaminergic system (12). For example, Kunimatsu et al. (13) demonstrated that chronic hyperprolactinemia and maintained corpora lutea causing the decrease of bone density are commonly inducible in female rats undergoing long-term treatment with antipsychotics haloperidol and chlorpromazine (i.e., the DA D2 receptor antagonists). The affinity for DA D2 receptors, the penetration of the blood–brain barrier (BBB), and the dose required to adequately occupy cerebral D2 receptors play major roles in the hyperprolactinemic effects of antipsychotics and other xenobiotics (14). Serotonin (5-HT), which serves as an indirect modulator, also has a stimulatory role in PRL secretion in both the hypothalamus and the pituitary, probably mediated *via* the stimulation of PRL-releasing factors (15).

Olanzapine (OLZ), an atypical antipsychotic drug, has an intermediary binding affinity for DA D2 receptors, thereby inducing a moderate and dose-dependent elevation of PRL levels (15, 16). It also exerts antipsychotic effects and induces weight gain by blocking the 5-HT_2A_ and 5-HT_2c_ receptors, respectively (16, 17). Thus, the antagonism of OLZ toward the 5-HT_2_ receptor might partly explain its moderate PRL-elevating tendency (18, 19). In this regard, some gene polymorphisms in DA D2 and 5-HT_2A_ receptors (e.g., *DRD2* and *5-HTR2A*) have been found to affect PRL levels after OLZ administration (20). A logistic regression analysis of only 10 variables revealed that other risk factors, such as gender, dose, and fasting glucose levels, are also significantly correlated with elevated PRL levels in patients taking OLZ (21). Nevertheless, few studies have investigated the factors influencing PRL levels in OLZ-treated patients in light of multi-dimensional electronic health record (EHR) data. In addition, Wu et al. (19) reported that elevated PRL levels were significantly associated with sexual dysfunction in patients with schizophrenia who had received OLZ treatment. A previous study revealed the alterations in mitochondria of the rat spermatozoa after experimental hyperprolactinemia (22). Recently, an *in vivo* animal study by Khalaf et al. (23) demonstrated the role of ovarian mitochondrial dysfunction and oxidative stress in ovarian toxicity induced by antipsychotics. On the other hand, the findings by Chen et al. (24) revealed that changes in PRL levels in the course of OLZ treatment are closely correlated with improvement in positive symptoms of schizophrenia, indicating that the PRL level of serum is a useful biological marker for predicting the effectiveness of antipsychotics (25, 26). Hence, monitoring PRL levels during OLZ treatment is vital to minimize the risk of PRL-related adverse events and maximize the response to treatment by antipsychotics.

Interest in Artificial Intelligence (AI)-assisted pharmacovigilance has grown in recent years (27). Within the field of AI, machine learning (ML) is a data-driven computational methodology increasingly applied for predictions of the post-marketing side-effects of drugs (28). The EHR is a source of data for detecting such adverse drug reactions (ADRs) due to its advantages of housing a collection of accurate, detailed, and abundant information on patients (28, 29). For example, On et al. (30) developed ML models for eight types of chemotherapy-induced ADRs (e.g., the nausea–vomiting prediction model) by using EHR data. In addition, it has been demonstrated that ML algorithms allow for the prediction of responses to drug treatment (e.g., antidepressants and anti-cancer drugs) (31, 32) and disease outcomes (e.g., stroke) (33).

**Figure 1:**
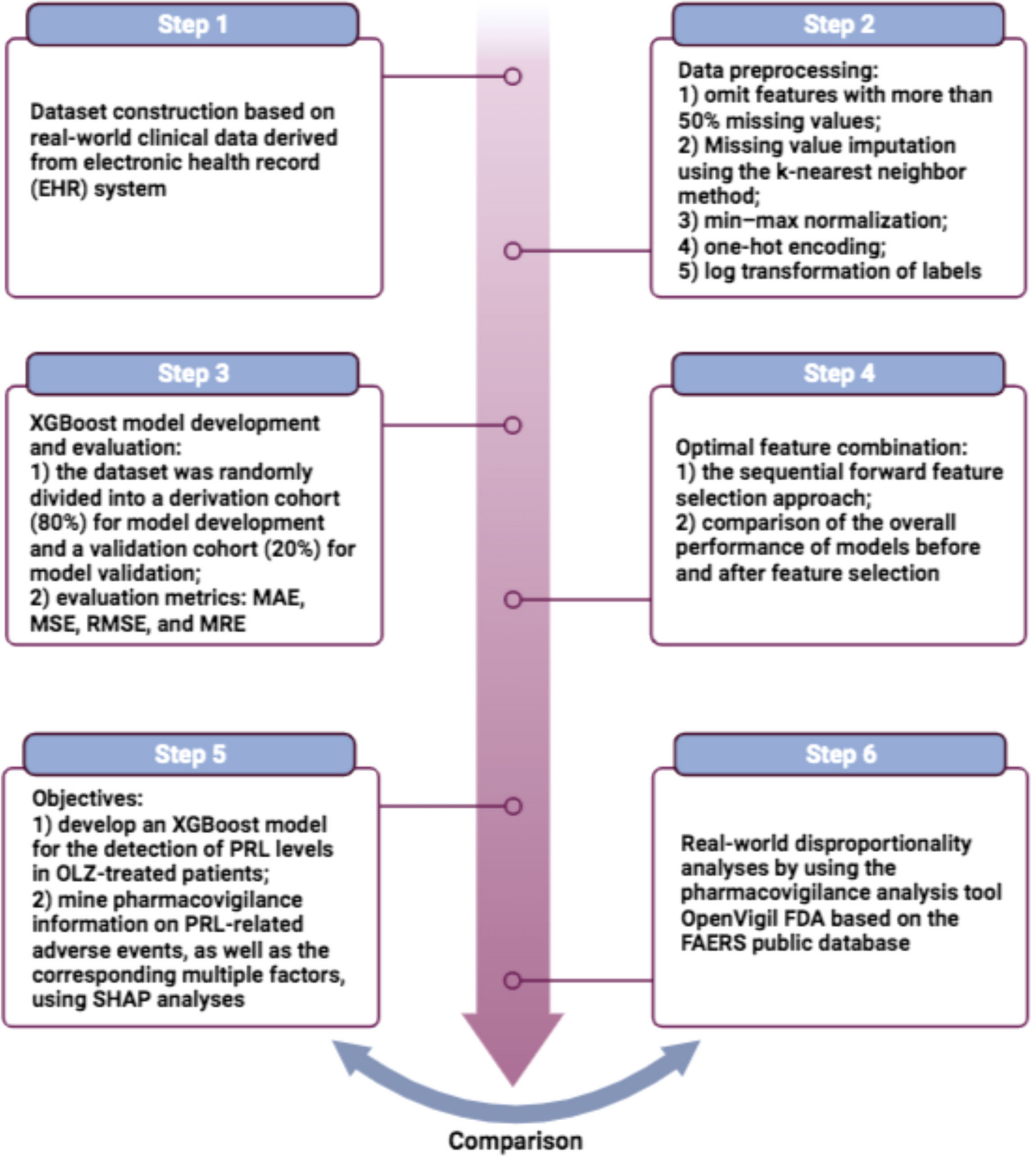
The flowchart of this work.

In this study, we use the eXtreme gradient boosting (XGBoost) algorithm, a well-known supervised ML algorithm widely used in medicine (34), to construct a model of PRL prediction associated with the side-effects and clinical effectiveness of OLZ by using EHR data. The objectives of this study are to i) develop an XGBoost model for the detection of PRL levels in OLZ-treated patients, and ii) identify multiple factors, particularly co-administered drugs, that may cause hypo- or hyperprolactinemia during OLZ treatment by using an interpretable ML method–the SHapley Additive exPlanations (SHAP) analysis (35). The results are then compared with evidence from real-world disproportionality analyses by using the pharmacovigilance analysis tool OpenVigil FDA (http://openvigil.pharmacology.uni-kiel.de/openvigilfda.php). This online tool uses the “openFDA” API of the US Food and Drug Administration (FDA) to access pharmacovigilance data from the FDA Adverse Event Reporting System (FAERS) (36). The flowchart of this work is shown in **Figure 1**.

## Materials and methods

### Data source

Clinical data on inpatients during OLZ treatment in the latter half of 2018 were mined from the EHR system of the Affiliated Brain Hospital of Guangzhou Medical University in China. The independent ethics committee of the hospital approved the data collection and waived the requirement of informed consent owing to the retrospective nature of our analyses ([2021] No. 027). We obtained 672 PRL measurements of 393 inpatients who had received OLZ treatment, along with information on the patients’ demographic characteristics, diagnoses, history of disorders, and combined medications and biochemical analyses that were determined at the same time points as their PRL levels. Finally, 473 features were identified that, along with the label–PRL, formed the dataset for the ML tasks. A summary of these features is provided in **Table 1**.

**Table 1:** A summary of the features of patients taking olanzapine (OLZ) in our original data derived from the EHR system.

Items	Features
Demographic characteristics (four features)	Gender, age, body weight (BW), height
History of disease and substance abuse (four features)	Allergic history, smoking history, drinking history, history of other substance abuse
Diagnoses (six features)	Diagnosis of schizophrenia, diagnosis of bipolar affective disorder, diagnosis of diabetes, diagnosis of hyperlipemia, diagnosis of hyperuricemia, diagnosis of hypertension
Blood types, phenotypes, genotypes, and gene polymorphisms (15 features)	ABO blood type, Rh blood type, *CYP2C19* phenotype, *CYP2D6* genotype, *CYP2D6* phenotype, *ApoE* genotype, *MTHFR* phenotype, HLA-B*1502 genotype, *CYP2C19* genotype, *MTHFR (C677T)* polymorphism, *CYP2D6 (G4180C)* polymorphism, *CYP2D6 (G2988A)* polymorphism, *CYP2D6 (C2850T)* polymorphism, *CYP2D6 (G1846A)* polymorphism, *CYP2D6 (C100T)* polymorphism
Information on OLZ (one feature)	Daily dose of OLZ
Co-administered medications (280 features)	1) Western Medicine: risperidone, diazepam, oxcarbazepine, ceftriaxone, nimodipine, duloxetine, ganciclovir, loratadine, metoprolol, etc. 2) Traditional Chinese Medicine: Shugan Jieyu capsules, Shuxuening injection, Shedan Chenpi powder, Jinshuibao tablets, Jiuwei Zhenxin granules, etc.
Biochemical analyses (163 features)	1) Blood routine examination: white blood cell count (WBC), absolute monocyte count (MONO#), platelet distribution width (PDW), etc. 2) Therapeutic drug monitoring: Concentrations of OLZ (C_OLZ), risperidone (C_ Risperidone), sertraline (C_Sertraline), fluoxetine (C_Fluoxetine), fluvoxamine (C_Fluvoxamine), venlafaxine (C_Venlafaxine), lamotrigine (C_Lamotrigine), aripiprazole (C_Aripiprazole), etc. 3) Electrolytes: serum sodium (Na), serum potassium (K), serum phosphorus (P), etc. 4) Hepatic and renal function: alanine transaminase (ALT), aspartate aminotransferase (AST), serum creatinine (Cr), total bilirubin (TBIL), etc. 5) Others: Total cholesterol (TC), creatine kinase (CK), C-reactive protein (CRP), uric acid (UA), testosterone, α-L-fucosidase (AFU), and lipoprotein (a) [Lp(a)], cortisol, prolactin (PRL), progesterone, thyroxine, etc.

### Data preprocessing

Data preprocessing is vital for acquiring high-quality data for modeling. We first omitted the features that had more than 50% missing values and then imputed those with fewer than 50% missing values by using the k-nearest neighbor method (37). Subsequently, min–max normalization and one-hot encoding were applied to the continuous variables and the categorical variables, respectively. Finally, the labels were transformed into the logarithmic scale.

### Model development and evaluation

The final dataset in 672×384 matrix format was generated after data preprocessing, and was subsequently randomly divided into a derivation cohort (80%) for model development and a validation cohort (20%) for model validation. The XGBoost algorithm with the default hyperparameter settings was chosen for the regression prediction task. The metrics used for model evaluation were the mean absolute error (MAE), mean squared error (MSE), root-mean-squared error (RMSE), and mean relative error (MRE) (%). They are defined as follows:


$$MAE=\frac{1}{n}\;\sum_{i=1}^{n}\left|y_{i}-{\hat{y}}_{i}\right|$$

$$MSE=\frac{1}{n}\;\sum_{i=1}^{n}{\left(y_{i}-{\hat{y}}_{i}\right)}^{2}$$

$$RMSE=\sqrt{\frac{1}{n}\;\sum_{i=1}^{n}{\left(y_{i}-{\hat{y}}_{i}\right)}^{2}}$$

$$MRE\;\left(\%\right)=\frac{1}{n}\;\sum_{i=1}^{n}\frac{\left(y_{i}-{\hat{y}}_{i}\right)}{{\hat{y}}_{i}}\times 100\%$$

Where *y_i_* and ${\hat{y}}_{i}$ are the predicted and the actual values, respectively.

### Optimal feature combination and interpretation

Redundant and irrelevant features can increase the computation time, and negatively impact and reduce the learning accuracy of the models. This problem can be solved by feature selection (38). We used the mean absolute SHAP values (|SHAP value|) to illustrate the global importance of features (39). The sequential forward feature selection approach was then employed to generate the optimal feature subset (40). The general practices in this bottom-to-top search method involved starting with an empty feature subset, adding one feature out of the remaining features in each iteration (the order of addition depended on the feature importance calculated by the SHAP values: the more important the feature was, the greater was the precedence it had), and then evaluating the pros and cons of the generated feature subset by using 10-fold cross-validation on the derivation cohort. The optimal feature combination was obtained when “no considerable alteration” of the MAE values was observed in the test sets. Subsequently, SHAP plots were drawn to interpret the contributions of these features to the outputs of the model. Finally, the overall performance of models before and after feature selection was compared on the validation cohort by using the abovementioned metrics.

### Disproportionality analyses of PRL-related adverse events with antipsychotics

We focused on co-administered medications that may have an impact on the PRL levels during OLZ treatment. To this end, the results of our SHAP analyses were compared with real-world evidence from disproportionality analyses based on the relative reporting ratio (RRR), a frequentist method, offered by OpenVigil FDA. The RRR was calculated as follows (36):


$$RRR=\frac{DE\times N}{E\times D}$$

where *N* denotes the total number of reports, *DE*, *E*, and *D* denote the numbers of reports when both the drug was used and the event occurred, the drug was used, and the event occurred, respectively.

We used the RRR to compare the strength of associations among a given list of antipsychotics (including OLZ, risperidone, sulpiride, amisulpride, aripiprazole, clozapine, quetiapine, ziprasidone, paliperidone, and perphenazine) with the adverse events “blood prolactin increased,” “hyperprolactinemia,” and “blood prolactin decreased.” The drug with the largest RRR value indicated the most proportional reporting of the reaction for it. Stopping the administration of this drug was thus considered first.

### Implementation

Data processing and modeling were conducted by using the libraries pandas, numpy, scipy, matplotlib, seaborn, missingno, sklearn, XGBoost, shap and palettable. All the ML tasks were implemented in Python by using the Jupyter notebook.

## Results

### Dataset overview

**Figure 2:**
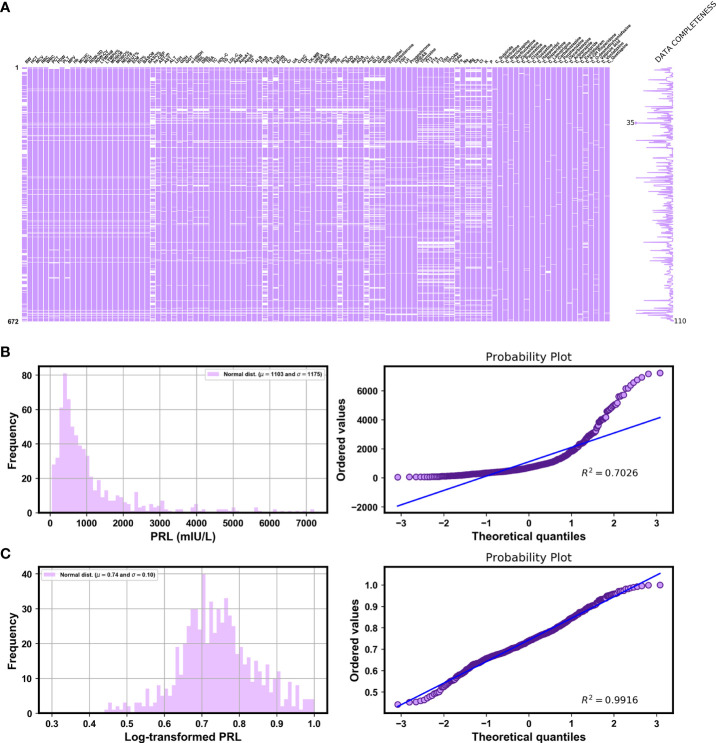
**(A)** Visualizing the missing data for features with fewer than 50% missing values by using the missingno library. Frequency histograms and quantile–quantile (Q–Q) plots of **(B)** PRL and **(C)** log-transformed PRL [calculated by log10(PRL)/log10(PRLmax)].

The final dataset consisted of 672 log-transformed PRL label values and 383 features (115 continuous features and 268 categorical features). **Figure 2A** shows the 110 continuous features with less than 50% missing values, represented by the white lines in each column. **Figures 2B, C** show the frequency histograms and quantile–quantile (Q–Q) plots of the labels before and after log-transformation, respectively. They indicated that the distribution of the log-transformed PRL ranging from 0.44 to 1 was the most symmetric and normal. **Table 2** shows the descriptions of the labels and partial features in our original data, without data preprocessing, derived from the EHR system.

**Table 2:** The descriptions of labels and partial features before data preprocessing.

Labels and partial features	Description (the total number of input-output data pairs is 672)	
Categorical data	Values	Distribution [n (%)]
Gender	Male	360 (53.57%)
Diagnosis of schizophrenia	Yes	327 (48.66%)
Smoking history	Yes	90 (13.39%)
Risperidone	Yes	101 (15.03%)
Paliperidone	Yes	6 (0.89%)
Amisulpride	Yes	20 (2.98%)
Sulpiride	Yes	24 (3.57%)
Aripiprazole	Yes	36 (5.36%)
Continuous data	Values [median (min–max)]	Missing [n (%)]
PRL (mIU/L)	708.19 (50.38–7216.28)	0 (0%)
Age (years)	45 (12–91)	0 (0%)
BW (kg)	61.35 (37–104)	178 (26.49%)
Daily dose of OLZ (mg)	15 (1.25–30)	0 (0%)
ALT (U/L)	18 (3–399)	24 (3.57%)
C_Aripiprazole (ng/mL)	0 (0–647.37)	5 (0.74%)
C_OLZ (ng/mL)	30.44 (2.14–127.31)	0 (0%)
Progesterone (ng/mL)	0.6 (0.3–77.8)	31 (4.61%)
Testosterone (mmol/L)	8.74 (0.35–59.33)	31 (4.61%)
CK (U/L)	79.5 (16–1473)	48 (7.14%)
Na (mmol/L)	140.5 (121.0–146.5)	40 (5.95%)
P (mmol/L)	1.24 (0.62–2.90)	146 (21.73%)
AFU (U/L)	27.15 (6–70)	52 (7.74%)
Lp(a) (mg/L)	163.7 (3.2–1052.6)	56 (8.33%)
PDW (%)	15.8 (7.8–20.7)	41 (6.10%)

### Feature selection and interpretation

**Figure 3:**
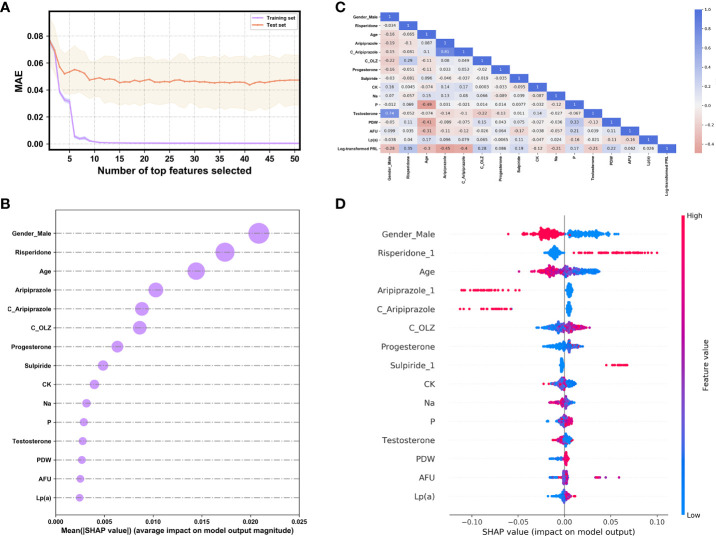
**(A)** The evolution of the performance of XGBoost models on the derivation cohort based on different compositions of the feature set. **(B)** The ranking by importance of the top 15 features according to the mean (|SHAP value|). **(C)** Heat map of the correlations between the log-transformed PRL and the selected features as analyzed by Pearson’s correlation coefficient. **(D)** The SHAP summary plot of the top 15 features. The red (blue) dots denote the high (low) values of the features. The high (low) SHAP values of the features denote their high (low) log-transformed PRL values.

**Figure 4:**
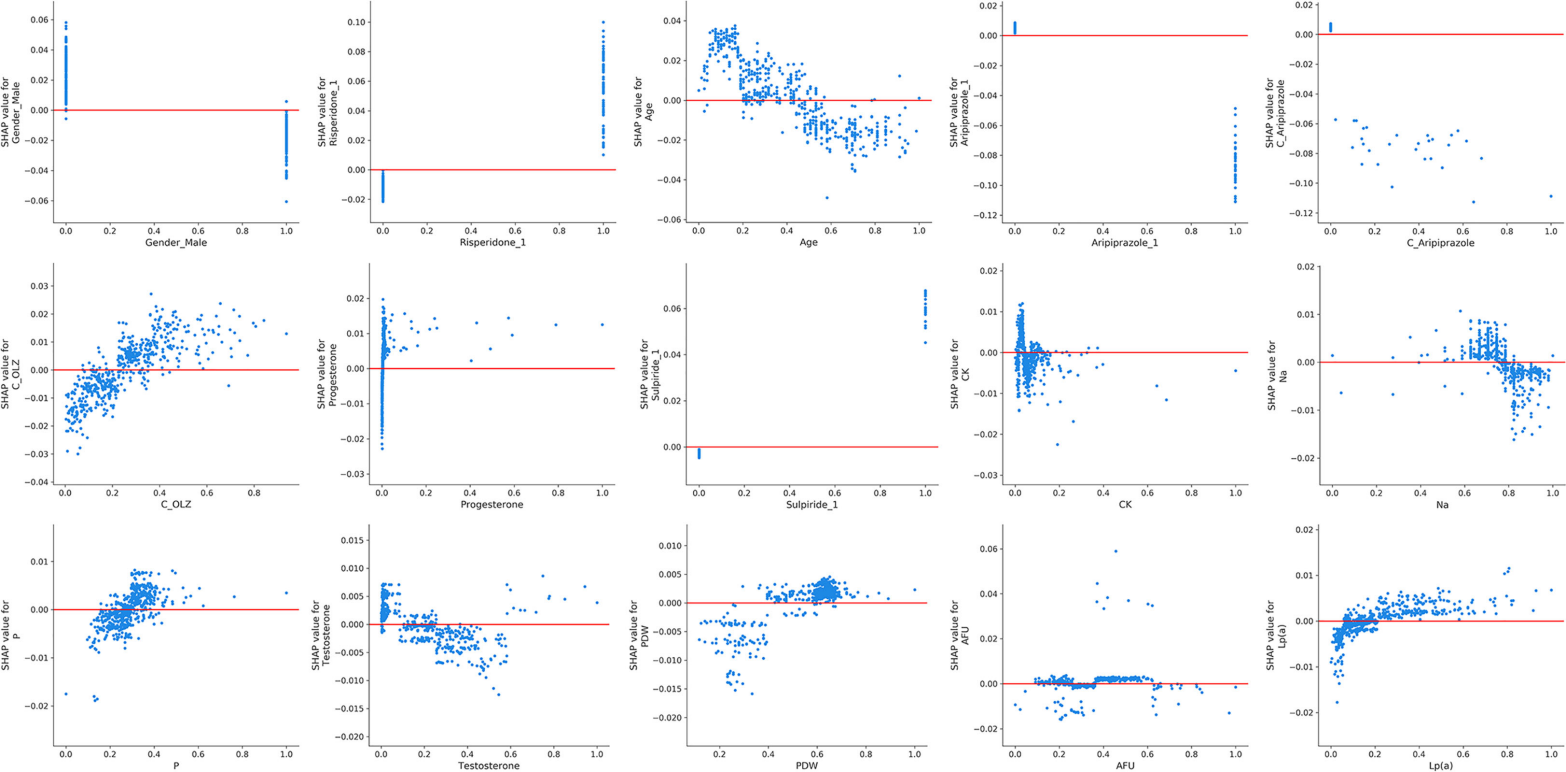
The SHAP dependence plots of the top 15 features. The SHAP values that exceeded zero represent high log-transformed PRL values, and vice versa.

**Figure 5:**
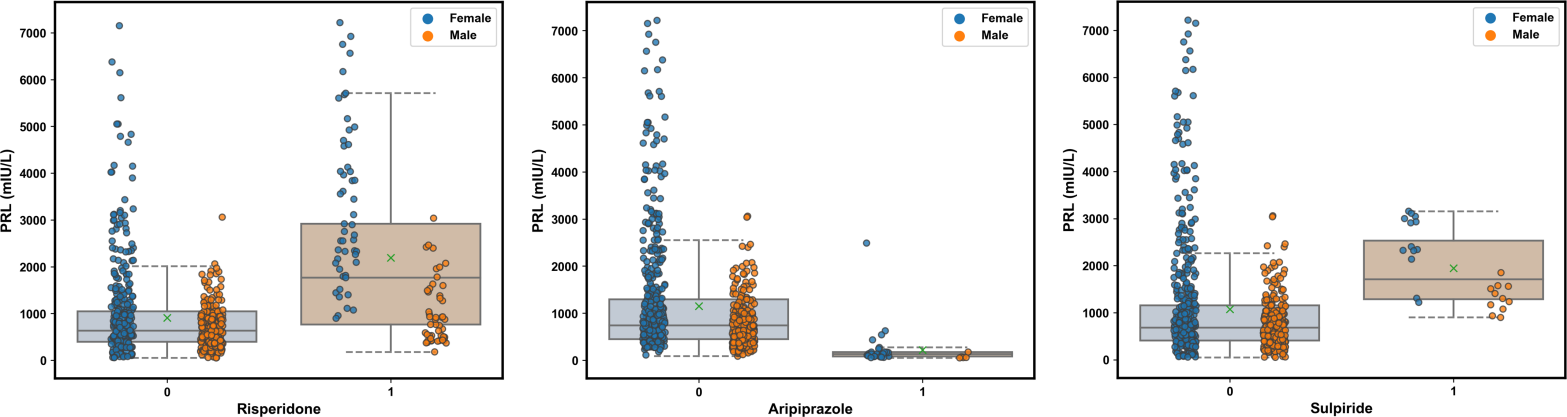
Comparisons of the influence of the co-administered risperidone, aripiprazole, and sulpiride on the PRL levels in terms of the gender of patients taking olanzapine (OLZ) according to the original data. The green multiplication sign indicates the mean PRL values.

**Figure 3A** presents the trend of evolution of the decline in the MAE in the training and test sets of the derivation cohort by using the forward feature selection strategy based on feature importance computed by using SHAP values. The top 15 features were identified as the optimal feature subset because the MAE declined imperceptibly with the addition of subsequent features. They were ranked according to the mean (|SHAP value|) as follows (**Figure 3B**): gender_male, co-administration of risperidone (Risperidone), age, co-administration of aripiprazole (Aripiprazole), concentration of aripiprazole (C_Aripiprazole), concentration of OLZ (C_OLZ), progesterone, co-administration of sulpiride (Sulpiride), creatine kinase (CK), serum sodium (Na), serum phosphorus (P), testosterone, platelet distribution width (PDW), α-L-fucosidase (AFU), and lipoprotein (a) [Lp(a)]. **Figure 3C** shows the Pearson’s correlations between the log-transformed PRL and these features, and indicates no prominent multi-collinear relationships among the features. **Figure 3D** presents the direction of effects of a variable on the output of the model. The SHAP dependence plots of these features show how they affected the outputs of our model (**Figure 4**). They indicate that higher PRL levels were related to females as well as the concomitant use of risperidone and sulpiride, and co-administered aripiprazole might have caused lower PRL levels. The comparisons of the influences of these three co-administered antipsychotics on PRL levels in terms of the gender of patients taking OLZ, based on the original data derived from the EHR system, are presented in **Figure 5**.

### Comparison of the performance of models

**Figure 6:**
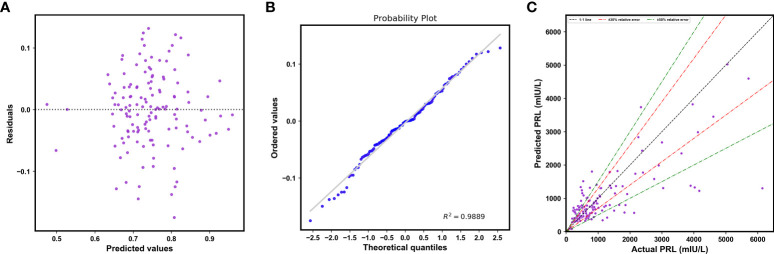
**(A)** Plot of residuals vs. the predicted log-transformed PRL values. **(B)** The normal plot of the residuals. **(C)** Scatterplot of the predicted PRL values vs. actual PRL values.

An overall comparison of the performance of models before and after feature selection is listed in **Table 3**. Among them, the XGBoost model after feature selection had better predictive performance, with lower values of the MAE, MSE, RMSE, and MRE. The lack of clear patterns and the symmetrical distribution of the residuals indicated that our proposed XGBoost model after feature selection was suitable for fitting the data in the validation cohort (**Figures 6A, B**). **Figure 6C** shows that 47.41% and 68.89% of the predicted values were within ranges of ±30% and ±50% of the actual values, respectively.

**Table 3:** Comparison of performance of XGBoost models on the validation cohort (N = 135) before and after feature selection.

XGBoost models	MAE	MSE	RMSE	MRE (%)
Before feature selection	0.046	0.0043	0.065	18
After feature selection	0.046	0.0036	0.060	11

### Disproportionality measures for antipsychotics and PRL-related adverse events

**Table 4** shows comparisons of the disproportionality measures for the 10 antipsychotics and PRL-related adverse events. Risperidone, paliperidone, and amisulpride were the top three antipsychotics associated with the risk of increased PRL concentration in the blood and hyperprolactinemia according to their RRR values. Similarly, aripiprazole exhibited the strongest association with decreased PRL content in blood, indicating that its use may protect patients taking OLZ from hyperprolactinemia.

**Table 4:** Relative reporting ratios (RRR) of prolactin (PRL)-related adverse events for a given list of antipsychotics.

Antipsychotics	Blood prolactin increased	Hyperprolactinemia	Blood prolactin decreased
Olanzapine	17.370	13.760	10.561
Risperidone	44.788	104.973	9.546
Sulpiride	NAN	NAN	NAN
Amisulpride	77.880	51.690	0
Aripiprazole	11.205	11.822	45.908
Clozapine	7.576	3.613	2.567
Quetiapine	8.487	6.665	2.185
Ziprasidone	23.303	10.334	5.697
Paliperidone	103.214	95.116	13.100
Perphenazine	7.838	16.499	0

Sulpiride is not approved for marketing in the United States by the FDA, and therefore was not found in the openFDA’s names of substances.

## Discussion

ML techniques have been successfully and widely applied to multiple fields in medicine, such as cancer diagnostics (41), treatment outcome predictions in patients with first-episode psychosis (42), and exploration of risk factors for the use of direct coercive measures in offender patients with schizophrenia spectrum disorders (43). AI-assisted pharmacovigilance has made large advancements but is still in its infancy. Chandak et al. (44) used ML to identify adverse drug effects posing increased risk to women based on public databases. We proposed a novel strategy that integrated ML with real-world clinical data of representative Chinese patient populations to advance AI-assisted pharmacovigilance studies. To the best of the authors’ knowledge, this is the first study to forecast the PRL level in OLZ-treated patients and mine pharmacovigilance information on PRL-related adverse events by integrating ML and EHR data. We used the XGBoost algorithm to construct an accurate model of PRL prediction that uses only two demographic characteristic predictors (i.e., gender_male and age), five predictors of drug information (i.e., risperidone, aripiprazole, C_Aripiprazole, C_OLZ, and sulpiride), and eight predictors of biochemical metrics [i.e., progesterone, CK, Na, P, testosterone, PDW, AFU, and Lp(a)]. This helps better understand these confounders that influence the PRL levels in OLZ-treated patients. They were found to be associated with the effectiveness of treatment and PRL-related side-effects.

The XGBoost algorithm, an ensemble learning method under the gradient boosting framework, is a scalable and distributed gradient-boosted decision tree ML library that allows for parallel tree boosting in both the classification and the regression tasks. It thus provides fast and accurate solutions for many problems in data science (45). It is generally considered to be a “black-box” model that loses the interpretability of the relationships between the inputs and the outputs of the models (46). In this study, we computed feature importance of the black-box XGBoost model by using the SHAP library, which used the SHAP values from game theory to estimate the contribution of each feature to the prediction in a model-agnostic manner (47). Furthermore, we drew more plots of interpretation, such as the SHAP summary plot and SHAP dependence plots, to show the general direction of influence and distributions of the SHAP outputs of each feature in the XGBoost model (48). In particular, our SHAP dependence plots demonstrated a non-linear relationship between C_OLZ and PRL, namely, a prominent trend of increase in the log-transformed PRL was observed as the normalized C_OLZ ranged from zero to approximately 0.4, and the trend of subsequent increase was not apparent. Moreover, we found that the log-transformed PRL was positively correlated with the range of the normalized C_OLZ from approximately 0.2 to 0.8 (corresponding to the C_OLZ ranging from approximately 25.03 ng/mL to 100.14 ng/mL). This range is close to the recommended therapeutic reference range (i.e., 20–80 ng/mL) and the laboratory alert level (i.e., 100 ng/mL) of OLZ according to the latest consensus-based guidelines of the *Arbeitsgemeinschaft für Neuropsychopharmakologie und Pharmakopsychiatrie* (AGNP)-*Therapeutic Drug Monitoring* (TDM) expert group (49). On the other hand, typical PRL levels with regard to the etiology of drugs are 25–100 ng/mL (approximately 530–2120 mIU/L) (50), which is broadly in line with our PRL measurements of OLZ-treated patients (see **Figure 2B**). Severe drug-related hyperprolactinemia (commonly defined as PRL values above 100 ng/mL), in the context of 100–250 ng/mL (approximately 2120–5300 mIU/L) and > 250 ng/mL (> ~5300 mIU/L), were found to occur in ~30% and ~5% of the cases (particularly with antipsychotics), respectively (50, 51). The aforementioned findings show that PRL may be a potential biological correlate for predicting the therapeutic effectiveness of OLZ and its PRL-related side-effects as well as their severity.

We also identified multiple confounders that might have influenced the PRL levels. They involved pathophysiological and pharmacological factors. The most important one was gender, i.e., females had more elevated RPL levels than males. A possible explanation for this is that females were more affected by antipsychotic-induced hyperprolactinemia than males (52). Likewise, our study revealed age-related changes in PRL levels, indicating that age is important because concomitant drug use and illnesses such as hypothyroidism and the degeneration of the ovarian secretion function, may be common in elderly populations (53, 54). Other hormones, including progesterone and testosterone, were also demonstrated to have effects on the PRL level. PRL commonly works antagonistically with estrogen and testosterone. It can inhibit the secretion of gonadotropin-releasing hormone by modulating the dopaminergic pathway, and thus may reduce testosterone levels associated with hypogonadism (55). It also acts directly on the granulosa cells of Graafian follicles to stimulate the release of progesterone and suppress estradiol production (56). The results indicated that hormone replacement (estrogen or testosterone) therapy may be an alternative pharmacological treatment strategy for hyperprolactinemia (57, 58). However, this strategy for antipsychotic-induced hyperprolactinemia is not recommended based on the latest evidence from network meta-analyses (59). PDW directly reflects the variability in platelet size, is considered to be an indicator of platelet activation and function, and is thus related to the extent of coronary artery disease (60). There are discrepancies in findings among some studies regarding the effects of PRL on platelet activation. Previous studies have demonstrated that hyperprolactinemia can cause adenosine diphosphate (ADP)-stimulated platelet activation, particularly in patients treated with antipsychotics. This might explain the increased risk for venous thromboembolism among them (61, 62). By contrast, Reuwer et al. (63) have suggested that PRL does not affect platelet aggregation or secretion in humans. Wahlberg et al. (64) found that PRL affected platelets in hyperprolactinemic patients in an indirect inhibitory way, indicating that it might have a protective role in thromboembolic disease. The positive correlation between PDW and the log-transformed PRL in our study suggests that hyperprolactinemia in OLZ-treated patients might be associated with the increased risk of thromboembolic events because PDW increases during platelet activation (65). Moreover, PRL may have direct effects on the metabolism of lipids—for example, reducing lipoprotein lipase activity in the human adipose tissue (66). Nevertheless, the positive correlation between the log-transformed PRL and Lp(a) in OLZ-treated patients, which has not been reported in any previous study to the best of our knowledge, indicates that increased cardiovascular risk in OLZ-treated patients with hyperprolactinemia should be the focus of research in this context, especially in males (5), as Lp(a) is an independent marker of cardiovascular risk (67). Our study also revealed the negative correlation between the log-transformed PRL and Na. This result is in line with that of a previous study suggesting that serum PRL may participate in sodium retention (68). CK, P, and AFU also played contributory roles in the log-transformed PRL levels, although few past studies have revealed the detailed mechanisms underlying their relationships. They possibly involve other physiological processes, such as bone mineral liberation and glycoprotein metabolism (69, 70).

In addition to pathophysiological factors, the concomitant use of risperidone, aripiprazole, and sulpiride were identified as the top three pharmacological contributors to the effects of PRL levels in OLZ-treated patients. These findings are largely consistent with our disproportionality analyses of PRL-related adverse events with antipsychotics, which suggest that risperidone should be discontinued first in OLZ-treated patients with hyperprolactinemia, and that the use of aripiprazole may protect them from this adverse event. Therefore, our study has highlighted the feasibility of AI-based pharmacovigilance detection in resource-limited settings by extracting various sources of data recorded in the EHR (71). Compared with OLZ and other atypical antipsychotics, risperidone is more likely to cause hyperprolactinemia owing to its incomplete crossing of the BBB, where this results in higher occupancy by DA D_2_ receptors in the pituitary gland than in the striatum (72). Compared to OLZ, risperidone demonstrated a more robust and persistent increase in PRL over a 24-h period in rats following acute and chronic administration (73). Aripiprazole is a partial agonist to the dopaminergic receptor D_2_, and has been endorsed by some guidelines for the treatment of antipsychotic-induced hyperprolactinemia (74, 75). Adjunctive aripiprazole or switching to aripiprazole in titration has been proved to be good PRL decrease effects (more than 50 ng/mL) for antipsychotic-induced hyperprolactinemia (59). Our work revealed gender-specific differences in these decreased effects (see **Figure 5**). Moreover, as a suitable alternative for OLZ-induced hyperprolactinemia, aripiprazole may not only diminish PRL levels but may also clear PRL-related symptoms that may occur with borderline or normal standardized PRL values while maintaining clinical stabilization (76). Notably, an abnormally low PRL level after switching to aripiprazole might occur, and this is a potential warning sign of a psychotic rebound. Routinely monitoring PRL levels may help avoid such a rebound (77). Eftekhari et al. (78) reported that oxidative stress and mitochondrial dysfunction played key roles in liver injury caused by OLZ, indicating that antioxidants, particular the nanoantioxidants involving increased bioavailability, stability, and target specificity (79, 80), were able to prevent OLZ-induced toxicity related to hyperprolactinemia (e.g., sexual dysfunction) (22, 23). Components of chamomile, a fascinating, well-known, and widely used medicinal plant, have effects on osteoporosis prevention, as well as the potent antioxidant, anti-inflammatory, and anti-cancer activities, which seem to be effective in the treatment of idiopathic hyperprolactinemia (75, 81). Therefore, antioxidants and herbal medications could be management options in OLZ-induced hyperprolactinemia when switching the antipsychotic is not an option.

Several limitations of this study should be noted. First, the small sample size might have affected the power of ML because the smaller the dataset is, the less powerful and less accurate are the results of ML algorithms (82). Thus, a larger number of samples is needed to improve the model generalization capability of the model. Second, although our findings were in accordance with previous reports demonstrating no influence of *CYP2D6* variation on PRL levels in antipsychotic-induced hyperprolactinemia (83), some candidate genes associated with changes in PRL were not included in our study. For example, *DRD2* may influence the susceptibility to hyperprolactinemia associated with OLZ treatment (84). Genetic associations of alterations in PRL in OLZ-treated patients may warrant further exploration. The confounding factors influencing the sex differences of pharmacovigilance signals on PRL-related adverse events and the pharmacogenetic mechanisms to explain these sex risks may be our future works.

## Conclusions

In this study, we constructed an ML-based model of PRL prediction in OLZ-treated patients by using the XGBoost algorithm and EHR data. Based on SHAP analyses, we also identified multiple pathophysiological and pharmacological confounders that influence PRL levels as tightly related to the effectiveness of treatment and PRL-related side-effects in OLZ-treated patients. Furthermore, our work suggests the feasibility of AI-based pharmacovigilance detection by using EHR as a source of data. In short, ML and EHR data can partner to facilitate the detection of PRL levels and pharmacovigilance signals in OLZ-treated patients.

## Data availability statement

The original contributions presented in the study are included in the article/Supplementary Material. Further inquiries can be directed to the corresponding authors.

## Ethics statement

The studies involving human participants were reviewed and approved by the independent ethics committee of the Affiliated Brain Hospital of Guangzhou Medical University. Written informed consent from the participants’ legal guardian/next of kin was not required to participate in this study in accordance with the national legislation and the institutional requirements.

## Author contributions

DS and YW together conceived and designed the study. XZ wrote the original draft preparation. TX and SH performed the data collection. JH conducted the data analyses. All authors contributed to the article and approved the submitted version.

## Funding

This work was supported by the Guangzhou municipal key discipline in medicine (2021-2023), Guangzhou Municipal Science and Technology Project for Medicine and Healthcare (grant numbers 20201A011047 and 20202A011016), Natural Science Foundation of Guangdong Province (grant numbers 2018A0303130074 and 2021A1515011325), Guangdong Provincial Hospital Pharmaceutical Research Fund (grant number 2022A22) and Science and Technology Plan Project of Guangdong Province (grant number 2019B030316001).

## Acknowledgments

We thank International Science Editing (http://www.internationalscienceediting.com) for editing this manuscript.

## Conflict of interest

The authors declare that the research was conducted in the absence of any commercial or financial relationships that could be construed as a potential conflict of interest.

## Publisher’s note

All claims expressed in this article are solely those of the authors and do not necessarily represent those of their affiliated organizations, or those of the publisher, the editors and the reviewers. Any product that may be evaluated in this article, or claim that may be made by its manufacturer, is not guaranteed or endorsed by the publisher.
